# Supplementary material for: Hormetic Effect of Glyphosate on the Morphology, Physiology and Metabolism of Coffee Plants
Source: Plants (Basel). 2023 Jun 8;12(12):2249. doi: 10.3390/plants12122249 (PMC10301858; doi:10.3390/plants12122249)
Supplement: Supplementary file 1 [file plants-12-02249-s001.zip › plants-2368535-supplementary.pdf]

Supplementary Material

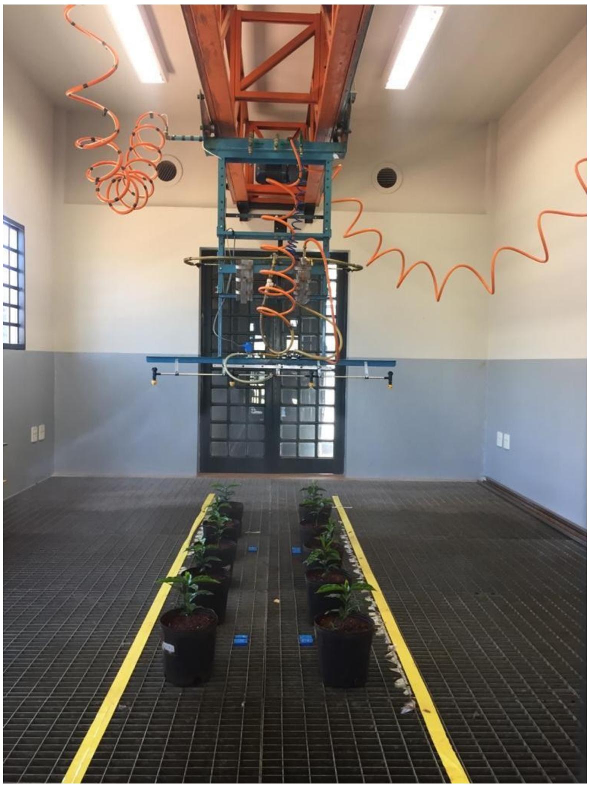

Figure S1- Application of glyphosate on coffee plants with an automated sprayer in a closed environment.

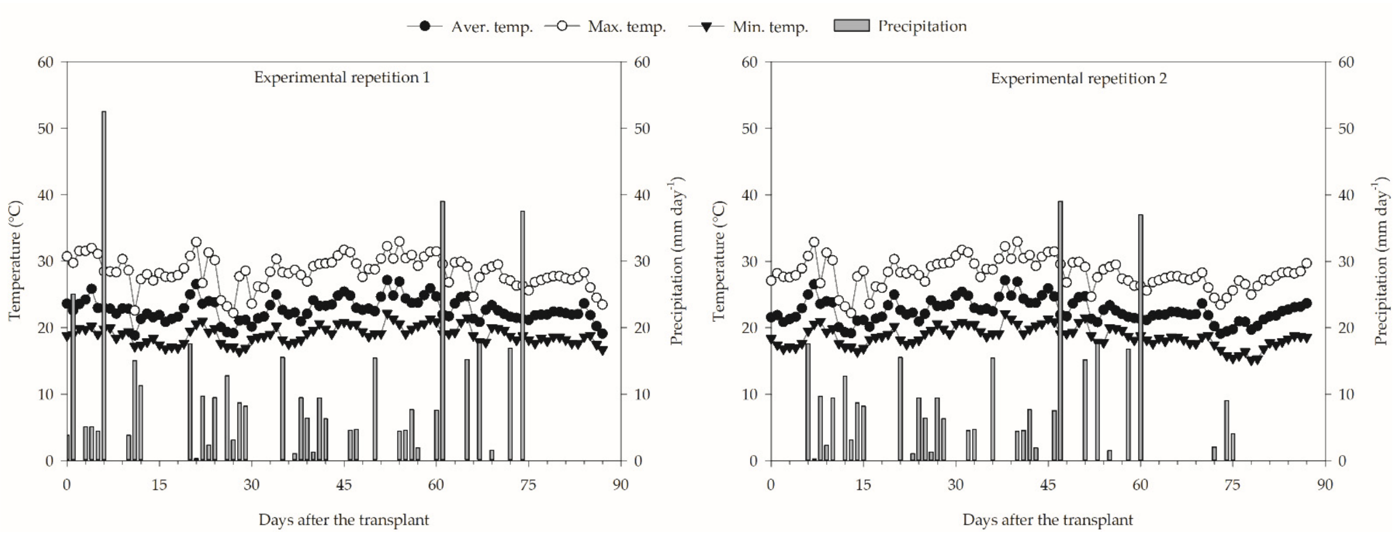

Figure S2- Average (Aver temp), minimum (Min temp) and maximum (Max temp) and daily precipitation (mm) temperatures after transplanting coffee seedlings.

Table S1. F values, coefficient of variation (CV), coefficient of determination (R^2), and constants of the regression equation for number of leaves and height of coffee plant at 7, 14, 21, 28, 35, and 42 days after application (DAA) of different doses of glyphosate

	Leaf number						Height					
	Days after application (DAA)											
	7	14	21	28	35	42	7	14	21	28	35	42
Doses	2.931**	10.009**	17.803**	18.725**	41.490**	66.855**	1.561^{ns}	6.507**	13.771**	22.745**	35.108**	41.030**
Hypothesis f≠0	-0.584	2.490	4.865	1.723	8.991	12.127	--	0.883	3.211	4.898	8.053	11.095
Model	2	2	1	2	1	1	--	2	2	1	1	1
Regression	7.88**	27.16**	38.15**	54.88**	91.62**	148.45**	--	18.03**	38.14**	49.74**	78.24**	90.82**
R^2	0.90	0.90	0.92	0.98	0.98	0.99	--	0.92	0.92	0.97	0.99	0.98
**Constants**												
b	0.59	0.66	1.27	1.53	2.36	2.98	--	0.80	0.84	1.36	1.42	1.65
c	12.55	12.30	9.85	13.48	13.34	13.38	--	13.80	13.19	12.47	12.06	14.41
d	16.72	18.54	20.31	22.44	23.00	26.73	--	21.43	23.90	24.35	25.76	26.99
e	1442.36	344.90	12.14	91.08	46.45	61.47	--	749.91	409.66	32.55	40.06	44.32
f	--	--	1.00	--	0.22	0.20	--	--	--	0.40	0.40	0.36
CV (%)	10.48	11.25	13.78	15.70	14.03	14.23	16.05	13.47	14.31	13.42	12.46	12.61

^{ns} not significant; **, * Significant at 1 and 5% probability by the f test, respectively.

Table S2. F values, coefficient of variation (CV), coefficient of determination (R^2), and constants of the regression equation for leaf (LDM), stem (SDM), and total (TDM) dry mass and leaf area (LA) of coffee plants at 42 days after application of different doses of glyphosate.

	LDM	SDM	TDM	LA
Doses	61.49**	22.23**	53.53**	97.74**
Hypothesis f≠0	23.34	8.57	21.778	31.50
Model	1	1	1	1
Regression	135.98**	48.11**	117.6**	217.92**
R^2	0.98	0.96	0.98	0.99
**Constants**				
b	1.49	1.14	1.42	2.14
c	1.94	-1.39	2.26	313.97
d	7.35	2.58	9.92	975.98
e	31.91	21.72	30.47	39.96
f	0.23	0.23	0.34	21.59
CV (%)	16.13	23.08	16.65	16.86

^{ns} not significant; **, * Significant at 1 and 5% probability by the f test, respectively

Table S3. F values, coefficient of variation (CV), coefficient of determination (R^2), and constants of the regression equation for CO_2 assimilation (A), transpiration (E), stomatal conductance (gs) and internal concentration of CO_2 (Ci), carboxylation efficiency (A/Ci), effective and intrinsic water use efficiency (EWUE and IWUE), and ETR/A ratio of coffee plants at 7, 21, and 42 days after application (DAA) of different glyphosate doses.

	**A**			**E**			**gS**			**Ci**		
	**Days after application (DAA)**											
	**7**	**21**	**35**	**7**	**21**	**35**	**7**	**21**	**35**	**7**	**21**	**35**
Doses	190.07**	182.77**	102.87**	78.83**	35.24**	29.07**	37.69**	45.86**	26.47**	89.80**	24.30**	25.42**
Hypothesis f≠0	55.32	164.59	106.36	49.00	21.17	12.05	56.61	27.99	8.31	--	--	--
Model	1	1	1	1	1	1	1	1	1	--	--	--
Regression	410.07**	400.01**	227.39*	155.52**	75.76	64.77**	79.01**	94.57*	58.26**	--	--	--
R^2	0.96	0.97	0.98	0.88	0.96	0.99	0.93	0.92	0.98	--	--	--
**Constants**												
b	1.13	1.18	1.83	1.09	1.14	2.12	1.32	1.30	1.96	--	--	--
c	-8.21	-7.85	0.87	-2.43	-0.89	0.79	27.70	32.97	25.87	--	--	--
d	5.04	5.85	4.28	2.81	2.66	2.03	66.07	72.72	53.31	--	--	--
e	15.94	12.06	28.26	5.01	10.45	32.28	3.43	14.13	32.06	--	--	--
f	1.03	1.77	0.29	1.30	0.44	0.06	28.55	4.06	1.34	--	--	--
CV (%)	14.02	14.80	18.86	10.83	17.05	28.14	14.92	10.57	22.38	7.17	17.15	18.47

	**A/Ci**			**EWUE**			**IWUE**			**ETR/A**		
	**7**	**21**	**35**	**7**	**21**	**35**	**7**	**21**	**35**	**7**	**21**	**35**
Doses	169.46**	14.31**	41.22**	103.29**	21.26**	1.80^{ns}	68.32**	96.77**	6.28**	35.16**	68.70**	21.45**
Hypothesis f≠0	-9.97	19.77	63.49	0.001	0.83	--	0.15	9.05	5.61	--	--	--
Model	2	1	1	2	2	--	2	1	1	--	--	--
Regression	482.94**	29.66**	91.06**	306.10**	58.76**	--	203.03**	205.35**	13.87**	--	--	--
R^2	0.95	0.92	0.98	0.99	0.93	--	0.99	0.94	0.98	--	--	--
**Constants**												
b	1.61	1.57	1.98	3.37	3.17	--	3.34	1.91	1.36	--	--	--
c	-0.0005	0.0004	0.0003	0.28	0.68	--	0.01	0.004	0.01	--	--	--
d	0.02	0.03	0.01	1.92	2.85	--	0.08	0.10	0.08	--	--	--
e	362.79	14.76	25.92	535.65	534.92	--	573.30	243.45	40.67	--	--	--
f	--	0.006	0.002	--	--	--	--	0.0003	0.003	--	--	--
CV (%)	15.62	64.84	42.49	15.25	29.69	53.08	18.84	16.90	39.82	53.77	48.33	46.19

^{ns} not significant; **; * Significant at 1 and 5% probability by the f test, respectively

Table S4. F values, coefficient of variation (CV), coefficient of determination (R^2), and constants of the regression equation for the electron transport rate (ETR) and effective photochemical efficiency of photosystem II (Φ_{PSII}) of coffee plants at 7, 14, 21, 28, 35, and 42 days after application (DAA) of different doses of glyphosate

	**ETR**						Φ_{PSII}					
	**Days after application (DAA)**											
	**7**	**14**	**21**	**28**	**35**	**42**	**7**	**14**	**21**	**28**	**35**	**42**
Doses	31.98**	29.29**	38.38**	18.05**	10.84**	36.90**	31.98**	29.29**	38.38**	18.05**	10.84**	36.90**
Hypothesis f≠0	9.11	10.03	0.56	9.76	12.13	15.55	8.079	10.02	-4.29	9.56	12.13	15.55
Model	1	1	2	1	1	1	1	1	2	1	1	1
Regression	69.28**	64.01**	109.25**	39.42**	23.68**	81.22**	69.02**	64.01**	80.73**	39.37**	23.38**	81.21**
R^2	0.96	0.97	0.95	0.97	0.97	0.98	0.96	0.97	0.95	0.97	0.97	0.98
**Constants**												
b	1.06	1.36	0.85	1.10	1.52	1.20	1.20	1.38	0.90	1.17	1.53	1.21
c	-148.70	30.17	-46.19	-2.26	34.26	14.89	-0.09	0.149	-0.10	0.09	0.16	0.07
d	44.11	65.82	62.78	53.61	45.54	59.51	0.21	0.313	0.30	0.26	0.22	0.28
e	75.13	42.15	10829.3	23.37	28.54	23.08	93.52	43.43	6717.30	28.67	28.83	23.33
f	2.76	1.09	--	2.88	0.87	2.52	0.004	0.005	--	0.01	0.004	0.01
CV (%)	15.55	12.34	8.21	10.09	12.99	10.69	15.55	12.34	8.21	10.09	12.99	10.69

^{ns} not significant; **, * Significant at 1 and 5% probability by the f test, respectively

Table S5. F values and coefficient of variation (CV) for the concentration of glyphosate, shikimic, ferulic, benzoic, and chlorogenic acids and of the aromatic amino acids tyrosine, phenylalanine, and tryptophan in coffee plants at 7, 21, and 42 days after application (DAA) of different doses of glyphosate

	**Glyphosate**			**Shikimic acid**			**Ferulic acid**			**Benzoic acid**		
	**Days after application (DAA)**											
	**7**	**21**	**42**	**7**	**21**	**42**	**7**	**21**	**42**	**7**	**21**	**42**
Doses	34,60**	43,39**	16,67**	50,83**	19,34**	7,26**	2,40*	3,98**	9,69**	5,14**	1,97^{ns}	0,76^{ns}
Hypothesis f≠0	--	--	--	--	--	--	--	--	--	--	--	--
Model	--	--	--	--	--	--	--	--	--	--	--	--
Regression	--	--	--	--	--	--	--	--	--	--	--	--
R^2	--	--	--	--	--	--	--	--	--	--	--	--
CV (%)	74,70	66,12	98,96	54,23	73,56	45,82	29,08	41,56	39,19	24,77	24,77	25,75
	**Chlorogenic acid**			**Phenylalanine**			**Tyrosine**			**Tryptophan**		
Doses	1,34^{ns}	0,96^{ns}	2,63*	6,58**	21,81**	5,33**	5,51**	65,68**	7,60**	10,72**	16,79**	2,01^{ns}
Hypothesis f≠0	--	--	--	--	--	--	--	--	--	--	--	--
Model	--	--	--	--	--	--	--	--	--	--	--	--
Regression	--	--	--	--	--	--	--	--	--	--	--	--
R^2	--	--	--	--	--	--	--	--	--	--	--	--
CV (%)	14,83	14,83	16,52	16,86	19,75	16,36	65,45	26,08	33,97	42,28	36,29	31,42

^{ns} not significant; **, * Significant at 1 and 5% probability by the f test, respectively

Table S6. F values, coefficient of variation (CV), coefficient of determination (R^2), and constants of the regression equation for the concentration of quinic, salicylic, caffeic, and coumaric acids in coffee plants at 7, 21, and 42 days after application (DAA) of different doses of glyphosate

	**Quinic acid**			**Salicylic acid**			**Caffeic acid**			**Coumaric acid**		
	**Days after application (DAA)**											
	**7**	**21**	**42**	**7**	**21**	**42**	**7**	**21**	**42**	**7**	**21**	**42**
Doses	5.18**	13.30**	31.27**	5.96**	3.84**	24.82**	3.31**	3.98**	4.15**	11.56**	9.24**	10.38**
Hypothesis f≠0	20.96	0.02	3.14	17.90	3.55	13.65	--	1.76	5.86	54.58	7.08	3.56
Model	1	2	2	1	2	1	--	2	1	1	1	2
Regression	11.36**	38.98**	91.37**	13.03**	9.70**	53.61**	--	11.17**	8.26**	24.82**	20.15**	28.57**
R^2	0.98	0.98	0.97	0.97	0.84	0.96	--	0.94	0.88	0.95	0.97	0.92
**Constants**							--					
b	1.79	1.08	1.87	1.72	1.30	1.21	--	4.31	2.57	1.92	2.02	1.16
c	1516.43	1834.97	428.60	0.38	0.55	-0.31	--	4.13	2.27	1.55	1.39	1.07
d	1672.73	5115.95	2431.60	0.55	1.36	0.63	--	8.66	3.95	1.75	6.14	3.47
e	4.24	306.53	197.37	10.20	518.61	7.30	--	379.45	224.21	2.88	85.15	220.91
f	1136.21	--	--	0.18	--	0.20	--	--	0.02	5.82	0.11	--
CV (%)	30.90	18.72	22.62	44.79	31.07	26.19	32.28	34.08	31.26	38.32	38.40	26.49

^{ns} not significant; **, * Significant at 1 and 5% probability by the f test, respectively
